# 

**Published:** 1851-09

**Authors:** 


### CONTENTS:

### PART I.—ORIGINAL COMMUNICATIONS.

ART. 1.—Clinical Lectures in the Medical Wards of the Illinois General Hospital, by Prof. N. S. Davis, - - - - - - - - - 162
" 2.—An Experimental Inquiry concerning some points in the Vital Processes of Assimilation and Nutrition, by Prof. N. S. Davis, - - 169
" 3.—A Case of Sciatica treated successfully by Inoculation with Sulphate of Morphine, by Charles Brackett, M. D., - - - - 190
" 4.—External Application of Chloroform in Erysipelas, by A. L. Castleman, M.D. 192
" 5.—Extraction of Child by a Novel Precess, by H. E. Ames, M. D., - 193
" 6.—The Uses of the Chloride of Sodium, by H. A. Johnson, - - 193
" 7.—On the Use of Cod Liver Oil in Nursing Sore Mouth, by Prof. Jno. Evans, 199
" 8.—Address delivered before the Ill. State Med. Society, by Prof. Herrick. 202
" 9.—Case of Puerperal Convulsions, by J. A. Preston, M. D., - - - 216
" 10.—Cass County, (Mich.) Medical Society, - - - - - - 219

### PART II.—REVIEWS AND NOTICES OF NEW WORKS.

ART. 1.—Experimental Researches, by Bennet Dowler, M. D., - - 220
" 2.—American Medical Association, - - - - - 224

### PART III—EDITORIAL.

ART. 1.—Western Medico-Chirurgical Journal, - - - - 228
" 2—Pure Drugs and Medicines, - - - - - 229
" 3 & 4.—To the Medical Profession, - - - - - - 230
" 5.—Miscellaneous Medical Intelligence, - - - - - 231
" 6—Notice to Readers and Correspondents. Obituary. - - 233

